# Thigh muscle co-activation during a single leg hop for distance one year after ACL-reconstruction and its relationship with a 2D knee flexion score: a cross-sectional study

**DOI:** 10.1186/s12891-026-10236-x

**Published:** 2026-07-30

**Authors:** Jano Probst, Patric Eichelberger, Aglaja Busch

**Affiliations:** Department of Health Professions, Physiotherapy, Bern University of Applied Sciences, Murtenstrasse 10, Bern, CH-3008 Switzerland

**Keywords:** Anterior cruciate ligament reconstruction, ACL, Return to sport testing, Single leg hop for distance, Neuromuscular control, Electromyography, Co-activation, Co-contraction index, 2D movement analysis

## Abstract

**Background:**

Landing alterations during single leg hop to distance (SLHD) testing persist after anterior cruciate ligament (ACL) reconstruction and may contribute to a high risk of re-injury. Increased thigh muscle co-activation is recognised as a common neuromuscular adaptation after ACL injury, but conflicting results have been reported for jump landings. Therefore, this study aimed to assess changes in co-activation at one year post-reconstruction during the landing of a SLHD and to evaluate whether a 2D knee flexion scoring system could identify landings with heightened co-activation.

**Method:**

This cross-sectional study assessed 18 sportingly active individuals one year after ACL-reconstruction alongside 18 matched control participants. Co-activation of the quadriceps and hamstring musculature during landing was recorded via electromyography. Additionally, peak knee flexion was assessed and scored as < 60°, between 60° and 80 ° and > 80° with a clinically practical 2D analysis. Co-activation was compared between the ACL-reconstructed leg, the contralateral leg and the matched control leg as well as between 2D-scored trials with statistical parameter mapping (SPM).

**Results:**

SPM analysis revealed greater thigh muscle co-activation in ACL-reconstructed leg compared to the matched control leg from 229 ms to 250 ms after initial contact (*p* = 0.007). No significant differences were observed between the ACL-reconstructed and contralateral leg. 2D knee flexion scoring did not reveal differences in co-activation during landing.

**Conclusion:**

Neuromuscular control is not fully restored at one year post-ACL-reconstruction, and consideration of thigh muscle co-activation during single leg hop testing could be important. However, a 2D knee flexion scoring system is not suitable for revealing co-activation differences among recreational sportingly active people, and alternative methods are needed to assess these neuromuscular adaptations in the clinic.

**Trial registration:**

The study was pre-registered at the German Clinical Trials Register (DRKS-ID: 00023002).

**Supplementary Information:**

The online version contains supplementary material available at https://doi.org/10.1186/s12891-026-10236-x.

## Background

Although surgical reconstruction of the anterior cruciate ligament (ACL) aims to restore the mechanical stability of the knee [1, 2], persistent knee symptoms, high re-injury rates, and an increased risk of post-traumatic osteoarthritis remain major concerns [3–5]. It is assumed that this results from an altered somatosensory perception, modifications in the central nervous system and alteration in motor output [6]. This altered motor output has been seen to manifest as biomechanical differences and alteration in neuromuscular function during gait, jumping, and landing for several years after ACL reconstruction [7–9]. Consequently, many ACL-reconstructed individuals appear to return to activity without complete functional recovery, despite efforts to improve postoperative rehabilitation through criterion-based decision making [10–13]. From a rehabilitation perspective this suggests that current return-to-sport tests or rehabilitation discharge criteria are insufficient or, at least, require a better understanding [10, 14, 15].

The single leg hop for distance (SLHD) is a widely used test during ACL rehabilitation and return to sport assessments [10, 16]. In recent years, kinematic and kinetic studies questioned the use of hop distance but highlighted the importance of assessing SLHD landings [16, 17]. While there is a substantial amount of data on biomechanical changes, relatively few studies have used electromyography (EMG) to investigate the SLHD [16]. EMG has been advocated and widely used to assess neuromuscular control after ACL-reconstruction, with numerous outcome measures reported in the literature [18, 19]. One of these outcome measures is co-activation, which is widely used in ACL research of the thigh muscles due to its sensitivity to changes in motor control and its direct effect on joint loading mechanics [18, 20]. Although different computational approaches have been used in the literature to characterise co-activation they generally aim to capture the interaction between agonist and antagonist muscles, which cannot be inferred from single-muscle EMG amplitudes alone [18, 21]. In the present study, thigh muscle co-activation is understood as the magnitude of simultaneous quadriceps and hamstring activity, taking into account both the relative activation and the overall activity of the two muscle groups, and is interpreted as an expression of neuromuscular coordination around the knee [22]. Co-activation is a subject of considerable interest, given its proposed role in enhancing knee stability by increasing joint-stiffness during dynamic movements, thus reducing rotational and shear forces which could protect the ACL [23–25]. However, excessive co-activation of muscle pairs can also reduce joint motion and decrease agonist force output leading to less efficient movement [23, 26].

Consistent with the proposed stabilising function, studies in individuals following ACL rupture and conservative treatment have reported increases in thigh muscle co-activation [22, 27]. This could indicate that individuals following ACL rupture may rely on heightened co-activation to compensate for perceived instability. At the same time, greater co-activation has been associated with poorer knee function and reduced knee flexion during walking in this population [22, 27]. Furthermore, prolonged increases in medial knee muscle co-activation have been associated with greater progression of medial knee osteoarthritis, collectively suggesting that this neuromuscular strategy may also have adverse functional consequences [28].

A similar increase in co-activation of the thigh musculature has been observed in individuals following ACL rupture and surgical treatment during leg extension [29], walking [30], step down tasks [31] and stair ambulation [32] compared to uninjured control individuals. Moreover, the association of higher co-activation with less knee flexion excursion has also been shown for jump landings in uninjured as well as ACL-reconstructed individuals [33–35], thus linking increased co-activation to “stiff” landings, an often-observed landing adaptation after ACL reconstruction [36]. Overall, it could be hypothesised that increases in co-activation reflect a crude compensatory motor strategy in the absence of other appropriate dynamic stabilisation strategies, which may contribute to altered movement patterns and functional outcomes described in the literature [27, 30, 34].

Nonetheless, direct evidence regarding co-activation during landing after ACL-reconstruction is conflicting [34, 37–41]. Variability in landing tasks, co-activation measures, and time post-surgery, both within and between studies, makes these results difficult to interpret. Furthermore, these studies reported averages or integrals, offering limited information on the dynamic adaptation of co-activation throughout the landing. Consequently, to date, there is limited understanding on how thigh muscle co-activation is adapted after ACL reconstruction during the force absorption part of the SLHD.

Furthermore, given the limitations of EMG measurements outside research settings, there is a need for instruments to assess SLHD landings in clinical practice. Recently, it has been shown that sagittal knee kinematics can be assessed using 2D video analysis in a clinical setting [42, 43] and a knee flexion scoring system categorising SLHD landing has been proposed [44]. Building on the suggested link between co-activation and knee flexion, it could be hypothesised that sagittal 2D analysis holds promise for identifying landing strategies characterised by heightened co-activity. With this study, we therefore pursue two objectives. First, our primary research question addresses whether ACL-reconstructed individuals show differences in co-activation one year after reconstruction compared to their contralateral leg and healthy controls during the landing of a SLHD. Second, we aimed to explore if a scoring system based on a clinically friendly sagittal 2D movement analysis reveals differences in thigh muscle co-activation.

## Methods

### Participants

For this cross-sectional study, we assessed 18 individuals following ACL reconstruction who were recruited for a longitudinal observational study project through local clinic [45]. Those individuals had an ACL rupture confirmed by an experienced clinician through clinical examination and medical imaging and underwent ACL-reconstruction surgery. To reduce heterogeneity, only participants who had surgical repair with a quadriceps tendon graft were included. As part of the longitudinal study, these participants were invited at 12 months (± 2 weeks) after ACL-reconstruction for measurements where they performed a SLHD. Additionally, a control group of 18 healthy control participants was recruited through an advertisement at the university. The inclusion criteria for both groups were men and women aged between 18 and 50 years who were physically active for at least 2 × 45 min per week. Reasons for exclusion were as follows: previous ACL injuries, back pain or spinal diseases, muscular or neurological injuries/diseases, thrombosis or fractures in the last 12 months, tumours, vascular diseases, alcohol dependency or dementia, pregnancy, acute infection or “cold” and intensive physical training on the day before. The control group was matched for age, height, weight, sex, and leg dominance [46], defined as the preferred leg to kick a ball [47]. No further criteria were used for the matching. They had no other significant injuries to the leg in the last year. All participants provided written consent to participate, and the study was approved by the Kantonale Ethikkommission für die Forschung Bern (Approval No. 02200).

### Procedure

Participants anthropometric data (age, height and sex) were collected through a case report form and information on sporting participation was obtained using a custom questionnaire that was developed for this study (see Additional file 1 for an English version). Additionally, participants completed the Tegner Activity Scale (TAS) [48] as well as the Knee Injuries and Osteoarthritis Outcome Score (KOOS) [49]. To prepare participants for EMG measurements their skin was locally shaved, roughened through sandpaper and cleaned with alcohol. Electrodes (Type P-00-S, Blue Sensor^®^, Ambu, Denmark, interelectrode distance: 20 mm) were then placed on the vastus lateralis, vastus medialis, biceps femoris, and semitendinosus muscles according to the guidelines for the Non-Invasive Assessment of Muscles Project (SENIAM) [50]. For every muscle, the electrode impedance was controlled to a maximum of 2 kΩ (D175 Electrode Impedance Meter, Digitimer, Hertfordshire, UK). All participants then underwent a six-minute walking warm-up at 3 km/h on a treadmill (Quasar^®^ med, h/p/cosmos sports & medical GmbH, Nussdorf-Traunstein, Germany), equipped with two force transducers (Type KMB52, 10 kN, Megatron Elektronik AG & Co. KG, Putzbrunn, Germany) to detect the heel strike of each gait cycle. This procedure was later used for the submaximal normalisation of EMG data [51, 52].

For 2D kinematic analysis five reflective markers were placed on each leg. To estimate the joint centers of the hip, knee and ankle, markers were placed on the center of the greater trochanter, the lateral epicondyle, and the lateral malleolus, respectively. Furthermore, two tracking markers were placed on the middle of the thigh and the shank. Jump trials were performed on a wooden floor with an embedded force plate (AMTI, OR6) to detect initial contact (IC) while landing. A video camera (Blackfly S USB3*)* and a flashlight were positioned 1.9 m perpendicular to the centre of the force plate at a height of 0.5 m to record the landings at 120 Hz and ensure the reflection of the markers. Participants were filmed two times during a standing trial with the left and right legs facing the camera, which served to calculate offset angles between joint center- and tracking markers [42].

For the jump trials participants were asked to start on one leg, jump forward as far as possible, accelerate themselves with the movement of the arms to further land on the same leg and maintain their balance for 10 s. Participants were allowed multiple practice trials on each leg until they felt comfortable performing the jump. The starting leg was assigned by a randomisation list (http://www.randomization.com), and participants jumped until three successful trials were recorded [53, 54]. If participants landed outside of the force plate areas or lost their balance by stepping down with the contralateral leg or shifting the landing foot upon landing, the respective trial was not used for analysis. If necessary, the participants were encouraged to adjust the starting position to ensure they landed on the force plate area. After each trial, the jump distance was measured with a measuring tape on the floor.

### Data collection and processing

The EMG signals during treadmill walking were recorded with a Myon EMG 320 System (Myon AG, Schwarzenberg, Switzerland) at 4000 Hz. Signals were then converted from analogue to digital (NI PCI 6255, National Instruments^®^, Austin, USA; 1.25 MS/s, 16 Bit), registered and further processed with LabVIEW^®^-based software (Imago Record, Pfitec^®^, Endingen, Germany). The EMG signals recorded during the last minute of treadmill walking were full wave rectified, bandpass filtered at 10–500 Hz, and root mean squares (RMSs) were calculated. For each muscle, the mean activity over 30 gait cycles was calculated and used to normalise the EMG signal from each muscle during the subsequent jumping task. This normalisation method was adopted in our laboratory and was applied for prior measurements in the original longitudinal study [51, 52].

For jump landings the Myon EMG 320 was connected to Vicon acquisition hardware (Vicon Giganet, Vicon Motion Systems Ltd, Oxford, UK) and acquired with Vicon Nexus software at 1000 Hz (Vicon Motion Systems Ltd, Oxford, UK). The EMG signal was then processed with a custom-written code in MATLAB (R2022b, The MathWorks, Nattick, USA) [55, 56]. The raw signals were high-pass (10 Hz) and low-pass (400 Hz) filtered, smoothed with a moving RMS procedure of 100 ms window length [56] and normalised as described to obtain a linear envelope, which represents a commonly accepted EMG parameter for analysing time-dependent muscle activation in dynamic tasks [24]. To assess muscular activity during landing, we chose a time interval of interest from 100 ms before to 250 ms after IC. The 100 ms time frame was chosen because muscular pre-activation influences neuromuscular control of jump landings [19, 33, 57] and to ensure comparability with other studies [58]. A time frame of 250 ms after IC was used in similar studies [38–40]. Furthermore, it approximately corresponds with the time of maximal knee flexion in the SLHD and includes the time at which the highest knee forces are absorbed [58]. The IC was detected with a 10 N ground reaction force threshold from the force plate.

Thigh muscle co-activation was defined as the co-activity of the anterior (vastus medialis + vastus lateralis) and posterior (semitendinosus + biceps femoris) thigh muscles and was calculated according to the dynamic co-contraction index (CCI) of Rudolph et al. [22]: CCI= (EMGlow/EMGhigh) * (EMGlow+EMGhigh), where EMGlow is the activity of the less active muscle group and EMGhigh is the activity of the more active muscle group. This formula was applied to each data point of the obtained linear envelope EMG signals, resulting in a CCI time series. For analysis, the average of the three successful trials for each subject was used.

Sagittal knee kinematics were analysed with the Automated Video Analysis for Dynamic Systems (AVADS), an open source code that allows automated tracking and processing of digital markers by using an adjustable grayscale threshold [59]. AVADS has been shown to be reliable for assessing jump landings with good to excellent agreement with 3D motion capture [42]. An offset angle between the lines connecting both joint center markers (hip-knee and knee-ankle) and the lines connecting the tracking markers with the knee marker were calculated based on the standing trials. During jumping, only the two tracking markers and the marker on the knee were kept, and the offset angle was later added to obtain the anatomic knee angle [42]. AVADS was run on MATLAB (R2019a), where the markers were tracked, and their coordinates were filtered with a fourth-order recursive low-pass filter at 13 Hz [43]. When markers were occluded, the location was estimated via a spline interpolation in AVADS. For each jump trial, the peak knee flexion angle was exported and classified according to the three-point scoring system of Di Paolo et al. [44]. Landings were therefore categorised as peak knee flexion angles lower than 60°, between 60° and 80° and greater than 80° [44].

### Statistical analysis

Patient characteristics (age, weight, height, TAS score, KOOS Score, sporting activity) as well as average hop distance, were checked for normal distribution with the Shapiro-Wilk test and qq-plots. Descriptive statistics are presented as the means and standard deviations or medians and interquartile ranges. Differences between study groups were tested with an unpaired t-test for mean hop distance and a Mann-Whitney U-test for age, weight, height, KOOS, and TAS.

The primary outcome measure in this study was the CCI during landing, compared between the ACL-reconstructed leg, the contralateral leg and the matched control leg. To avoid reducing the CCI time series to integrals or a single average value, we used one-dimensional statistical parameter mapping (SPM) [60]. For SPM, all CCI time series were time normalised to 101 data points. Comparable to scalar t-tests, SPM calculates the test statistics at each time node. For inference statistics, a critical threshold is calculated by modelling the behaviour of random time-varying signals, and adjacent points that surpass this critical threshold are called “suprathreshold clusters” [61]. Cluster-specific p-values are then calculated through random field theory and represent the probability that random field processes with the same temporal smoothness would produce the respective suprathreshold cluster [60, 61]. SPM therefore accounts for the time dependence of EMG signals and has previously been applied to CCI time series [62, 63]. All analyses were conducted with the open-source code spm1d [64] in MATLAB (R2022b). Time series were assessed for normality in spm1d, revealing deviations from the normal distribution [65]. Parametric and nonparametric tests did not differ in terms of subthreshold clusters. However, as the p-values slightly differed, we decided to present the results from the nonparametric test. The CCI curves are presented as the mean and standard deviation regardless of distribution to enhance interpretability and comparability with other studies [58].

For group and leg comparisons, the average CCI curve over the three successful jump trials of each subject was used. A nonparametric two-tailed unpaired t-test was applied to compare the CCI between the ACL-reconstructed leg and the matched control leg, and a nonparametric two-tailed paired t-test was used to compare the injured leg to the contralateral leg. The significance level was set at alpha = 0.05 and Bonferroni corrected to alpha = 0.025 for both tests due to multiple testing. For comprehensiveness, we also analysed the normalised activity of each muscle separately. To test whether the three-point 2D knee flexion scoring system showed differences in co-activation, the CCI time curves of all 2D-scored trials, including both legs of the control group, were compared with a nonparametric one-way ANOVA with a critical threshold of alpha = 0.05 in spm1d. As those analyses are explorative, the results were not corrected for multiple comparisons.

If EMG data for one jump trial were missing or implausible upon visual inspection, the average of the other two trials was used for analysis. For the uninjured leg of one subject in the ACL-group, the EMG data were completely missing due to technical issues. This subject was excluded from the comparison between the injured and contralateral sides. If knee flexion data were missing due to missing markers or misplacement of the camera, the respective trials were omitted from the comparisons between knee flexion scores.

## Results

### Participant characteristics

In the group of 18 participants who underwent ACL reconstruction, only one individual presented with an isolated ACL injury. 15 participants with ACL reconstruction exhibited concomitant meniscal lesions at the time of injury. Furthermore, two participants sustained an additional medial collateral ligament injury with a meniscal lesion, and one participant had an additional concomitant chondral lesion to a meniscal lesion. There was a small, non-significant difference in the distribution of age, height, weight, and TAS between the ACL-reconstructed group and the control group. Participants one year after ACL-reconstruction had significantly lower total KOOS-scores and, on average, jumped significantly less distance on the injured leg in the SLHD than did the control participants (Table 1).

**Table 1:** Participant characteristics

Characteristic	ACL *N* = 18	Control *N* = 18	*p*-value
Age (years)*	24 (22–28)	27 (26–29)	0.07
Male: Female (n)	12/6	12/6	-
Leg Dominance right/left (n)	17/1	17/1	-
Height (cm) *	174.5 (168–178)	178.5 (167–184)	0.24
Weight (kg) *	69.3 (64–78)	74 (60–77)	0.81
TAS (max:10) *			
Pre-injury	5 (5–7)	-	-
Current*	5 (5–7)	5 (5–6)	0.875
KOOS Total (max:168)*	150 (146–156)	167 (166–168)	< 0.001
Hop-distance injured/matched (cm)	119.1 ± 26.7	141.1 ± 31.3	0.03
Hop-distance contralateral (cm)	130.4 ± 27.2	140.8 ± 28.4	0.27

Values are presented with mean ± SD for normally or median and interquartile range for non-normally distributed data. Independent sample t-test or Mann-Whitney U-test were used for between groups comparisons

*SD *Standard deviation, *ACL *Anterior cruciate ligament, *KOOS *Knee Osteoarthritis Outcome Scale, *TAS* Tegner Activity Scale

*Non-normally distributed data

### Co-activation between groups and legs

**Fig. 1:**
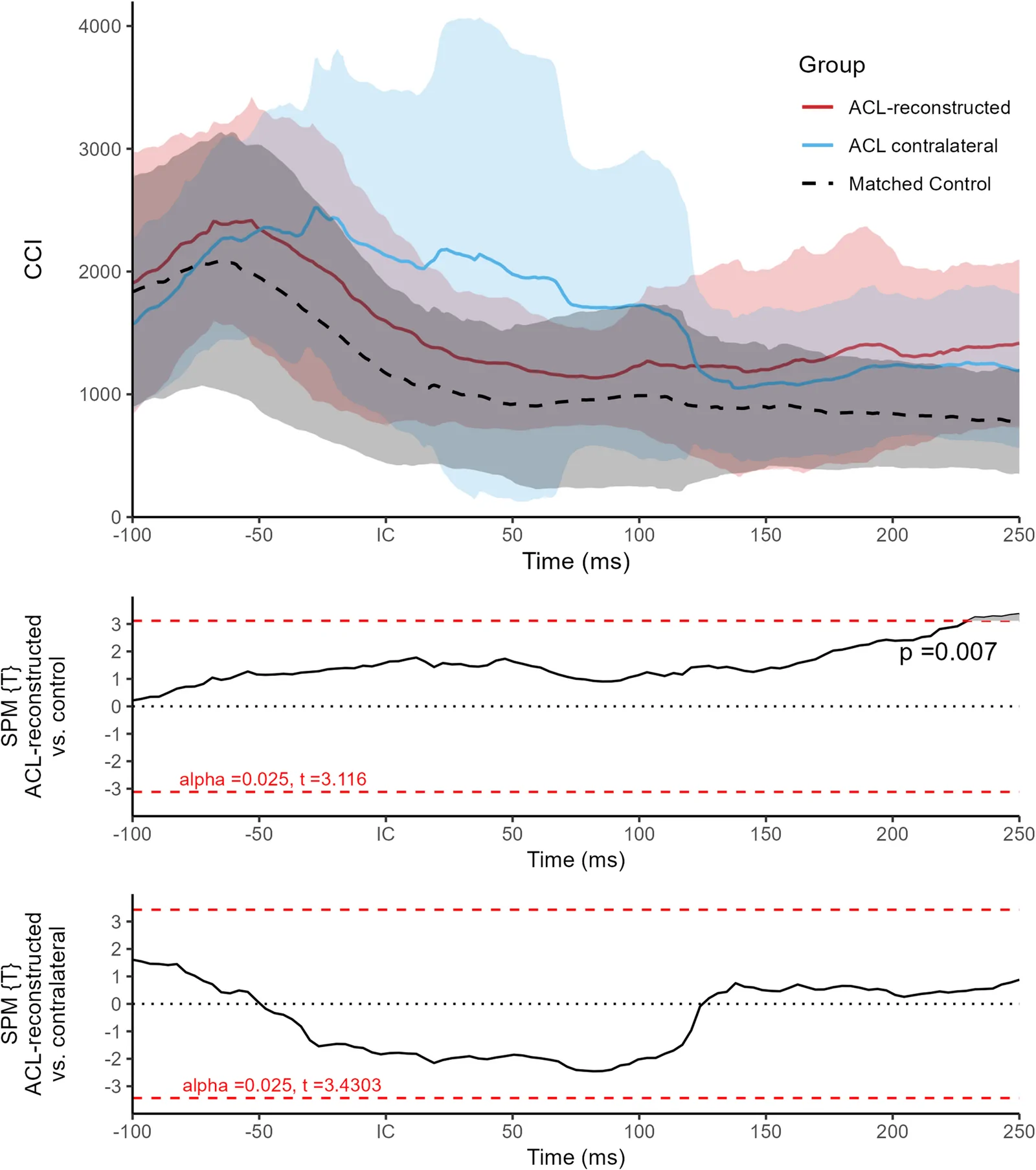
Co-activation between groups and legs. Upper row: average co-activation during landing for the ACL-reconstructed leg, the contralateral leg and the matched control leg. Shaded zones represent standard deviations. Middle row: SPM output of nonparametric unpaired t-test comparing ACL-reconstructed leg and matched control leg. Lower row: SPM output of nonparametric paired t-test comparing ACL-reconstructed leg and contralateral leg. If the t-curve (black line) exceeds the critical threshold (red dashed line), the difference is deemed significant. IC = Initial contact, ACL = Anterior cruciate ligament

In four participants, only two jump trials were included in the analysis of one side due to the presence of EMG artifacts in the semitendinosus muscle. The affected limb was the ACL-reconstructed leg in two participants, the contralateral leg in one participant, and the matched control leg in one participant. Analysis of the CCI time series demonstrated that co-activation during single leg hop landing was greatest approximately 75 ms to 25 ms before IC. Both groups revealed large within-group differences, with standard deviations reaching up to 72% and 78% of the mean values in the ACL-reconstructed leg and matched control leg, respectively. Standard deviations were accentuated within the contralateral leg, mostly due to two outliers (Fig. 1) The average CCI curves had similar shapes between the ACL-injured leg and the control group, with control participants landing on average with less co-activation during the whole analysed time frame. SPM analysis revealed only a small suprathreshold cluster between 229 ms and 250 ms after IC (94–100%), which exceeded the critical threshold of 3.12 (Fig. 1). The probability that this suprathreshold cluster would occur by repeated random sampling was 0.007. Therefore, the null hypothesis for this comparison was rejected. During the statistically significant time-period, the ACL-reconstructed leg had on average a 1.63-fold greater co-activation than the average control leg (CCI = 1393.6 ± 438.6 vs. CCI = 811.1 ± 419.9). Analysis of the CCI time series between the ACL-reconstructed leg and the contralateral leg yielded no significant differences (Fig. 1).

Analysis of individual muscles indicated a significant difference in normalised vastus medialis activity between limbs in the ACL group, with the contralateral leg showing greater normalised activity (Fig. 6 in Additional file 2). A suprathreshold cluster exceeded the critical threshold of 2.38 between 68 ms and 176 ms after IC (48–79%; *p* = 0.009). All other comparisons of individual muscles between the ACL-reconstructed leg and matched control leg or between the ACL-reconstructed leg and the contralateral leg demonstrated no significant differences (Additional file 2).

### Co-activation between 2D scored landings

**Fig. 2:**
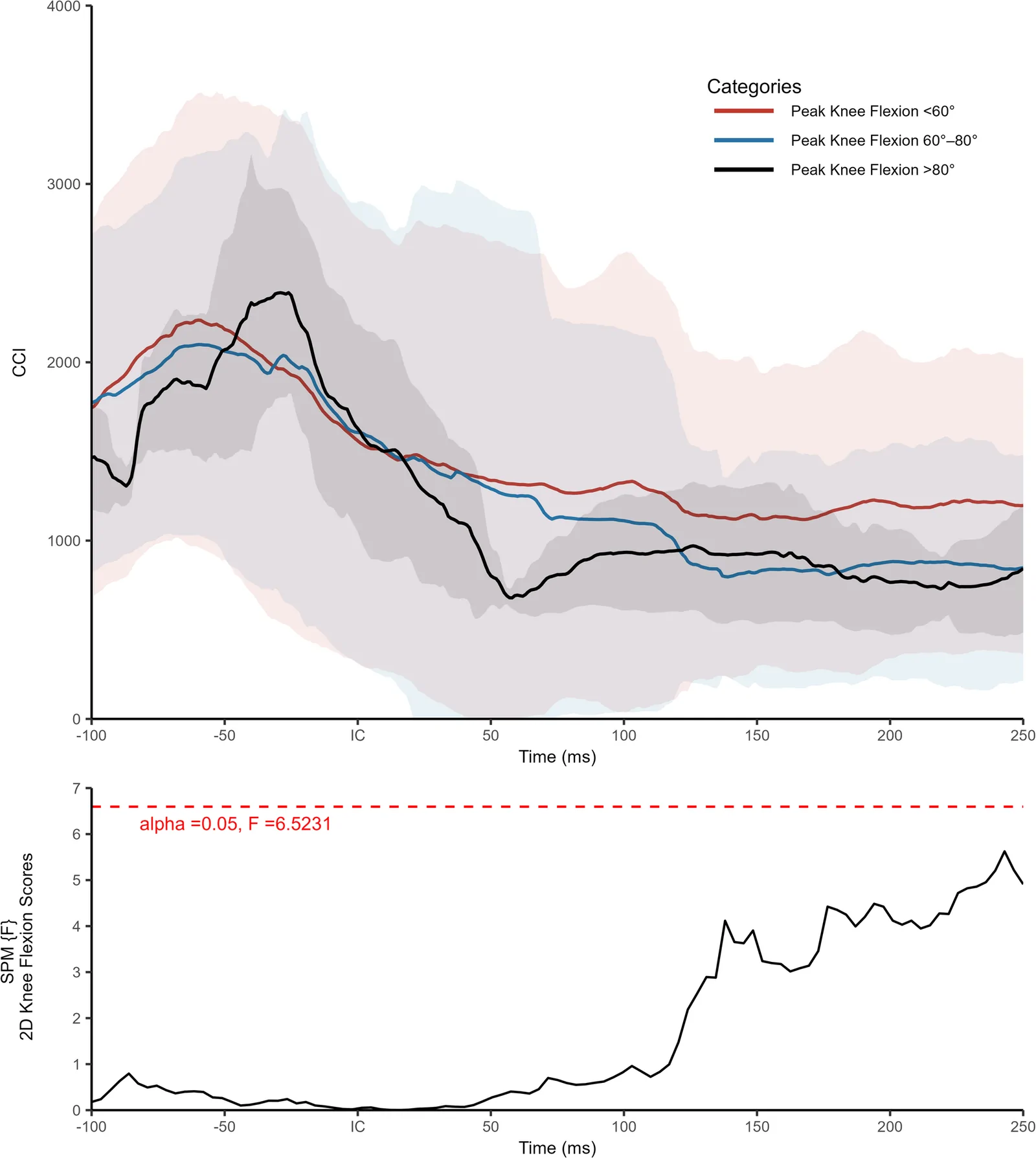
Co-activation between 2D-scored landings. Left: average co-activation during landing for 2D knee flexion categories. Shaded zones represent standard deviations. Right: SPM output of nonparametric one-way ANOVA comparing the three scoring-categories. If the f-curve (black line) exceeds the critical threshold (red dashed line), the difference is deemed significant. IC = Initial contact

A total of 216 jump landings were recorded, of which 29 were excluded from further analysis. Four jump landings were excluded due to EMG artefacts as previously described, and a further 25 due to issues with the video recordings (e.g. incorrect camera position, missing or occluded markers, or inappropriate lighting). Of the remaining 187 trials, 109 were scored in category 0 and 74 in category 1, while only four trials were scored in category 2 (Table 2). The peak knee flexion for the ACL-reconstructed leg, the contralateral leg and the matched control are presented in additional file 3. SPM analysis revealed no statistically significant differences in the CCI curves between the three categories (Fig. 2).

**Table 2:** 2D scored SLHD trials

	2D knee flexion category		
Group	0 [< 60°] (*n*)	1 [60–80°] (*n*)	2 [> 80°] (*n*)
ACL-reconstructed	42	5	0
ACL contralateral	25	21	0
Control	42	48	4

*SLHD*  Single leg hop to distance, *ACL*  Anterior cruciate ligament

## Discussion

This study aimed to assess changes in thigh muscle co-activation during the landing force absorption of a SLHD after ACL-reconstruction with a quadriceps tendon graft, specifically at one-year post-surgery, a time-point when individuals usually returned to full activity and were discharged from rehabilitation [66]. Additionally, we intended to determine whether changes in co-activation could be detected using a clinically practical 2D knee flexion assessment. We compared ACL-reconstructed participants one year after surgery with matched control participants and observed increased co-activation of the quadriceps and hamstring musculature in the ACL-reconstructed limb from 229 ms to 250 ms after initial contact. However, the 2D three-point knee flexion scoring system did not reveal any differences in co-activation.

### Co-activation between groups and legs

To our knowledge this is the first study to investigate changes in co-activation at a specific time point after ACL-reconstruction. Moreover, the use of the dynamic CCI by Rudolph et al. [22] and SPM analysis enabled us to assess differences throughout the landing, providing refined insights into co-activation changes. Consequently, these findings represent new evidence that physically active individuals post ACL-reconstruction use a motor strategy with greater co-activation, at least during the final phase of landing force absorption in the SLHD, when maximal knee flexion typically occurs [58].

From a neuromuscular control perspective, the observed increase in co-activation during the late landing phase may reflect an altered motor coordination strategy following ACL reconstruction. Co-activation of the quadriceps and hamstrings is considered to be a mechanism that enhances dynamic joint stability by increasing joint stiffness and limiting excessive tibial translation [23–25]. This may be particularly relevant in situations of high mechanical demand, such as jump landings [21]. In the context of ACL reconstruction, such a strategy may serve to compensate for persistent sensorimotor deficits, including impaired proprioception and altered afferent feedback, which have been reported after injury and surgery [6]. However, increased co-activation may also indicate a less refined motor control strategy and contribute to the “stiff” landing patterns commonly observed in this population [35, 36]. In the SLHD task, which requires both dynamic stability and effective force attenuation, these altered coordination patterns may therefore represent a trade-off between joint protection and movement efficiency. This interpretation is consistent with the reduced jump distance observed in the ACL-reconstructed group, which supports the notion that neuromuscular adaptations after ACL reconstruction may not be purely beneficial.

The increase in co-activation in the ACL-reconstructed leg aligns with observations from other dynamic tasks [29–32] and is supported by two studies that demonstrated increased co-activation during single-leg drop landings [40, 67]. In contrast, a recent meta-analysis, although with large heterogeneity, found no general differences in co-activation during landing in participants with ACL rupture compared to healthy controls [41]. In addition to differences in participant characteristics, landing tasks and landing phases, some methodological considerations may explain part of this discrepancy. For example, one study, which reported large opposite results, calculated an activity ratio based on non-normalised EMG signals [38], rendering group comparisons difficult to interpret [68]. Other studies operationalised co-activation with a hamstring-quadriceps ratio based on normalised EMG activities [37, 69]. It is important to consider that the ratio of total activity neither considers changes in the more active muscle over time nor accounts for the amount of activation. This could be important for its stabilising function [23, 69] and may explain differences in the results. However, the variation of results in the literature can also be interpreted in light of the small sample sizes in most studies and the inherent inter- and intraindividual differences in co-activation during dynamic movement [70]. We also found large interindividual differences, comparable to other studies assessing EMG activity during dynamic activities [9, 46, 58]. Additionally, differences in co-activation were only significant during a small proportion of the analysed landing phase. Therefore, the group-differences in this study and their implications must be interpreted with caution.

Furthermore, despite efforts to match the control group for several important characteristics, unaccounted differences could still have influenced the results. In particular, the reduced jump distance in the ACL group, accentuated on the reconstructed side, warrants attention as it could be a strategy to minimise landing demands [17]. Based on the available literature, it is not possible to predict in which direction this influenced co-activation results. Reduced landing forces could have altered the ratio of hamstring and quadriceps activation [71], but it is also plausible that reducing jump distance would conceal even larger differences in co-activation. In addition, the total KOOS score was significantly lower in the ACL-reconstructed group compared to the healthy control group, indicating a diminished knee function as reported by the patients. This difference may have influenced the present findings, as greater knee-related symptoms could potentially impact task performance. For instance, individuals with ACL reconstruction may have adopted a reduced jumping distance, as previously discussed, possibly impacting the observed neuromuscular patterns. Moreover, lower limb strength and range of motion were not assessed, which may have influenced the present findings. A similar limitation applies to the evaluation of subjective knee function. Although the TAS and the KOOS were used to provide a comprehensive characterisation of functional status, the inclusion of additional patient-reported outcome measures, such as the International Knee Documentation Committee (IKDC) score or the ACL-Return to Sport after Injury (ACL-RSI) scale, may have offered a more comprehensive assessment. Consequently, potential variability in these parameters could be indicative of unaccounted confounding factors.

By comparing the two legs of ACL-reconstructed participants, we observed a pronounced but non-significant increase of co-activation on the contralateral side shortly after IC. This was mainly driven by substantial outliers, manifesting in an accentuated dispersion in this time frame. While individual observations can be interesting, it is essential to refrain from overinterpretation of these findings. The most substantial outlier was observed in the individual with the greatest differences in jump distance between legs, which could explain part of the between-leg difference. However, the differences could also simply represent random variation. Therefore, our results are most plausibly interpreted as an indication of a mostly similar co-activation in both legs, adding to the body of evidence of bilateral neuromuscular changes after ACL-reconstruction [46, 72, 73]. The clinical relevance, role and function of increased thigh muscle co-activation are unclear, and several possible explanations for our findings exist. Still, the timing of the observed differences between the ACL-reconstructed leg and matched control participants in our study allow some interesting considerations.

First, thigh muscle co-activation is thought to increase knee stability [23–25]. In particular, hamstring co-activation can reduce anterior tibial shear force and the magnitude of ACL-loading during landing, as shown in simulation studies [74, 75]. This led to the suggestion that higher co-activation after ACL-reconstruction is a beneficial and protective adaptation [41]. ACL forces depend on a complex interplay of multiple factors [74], and muscle forces cannot be inferred from EMG activation alone [76]. However, given that anterior shear forces are highest during the first 100 ms after IC [74, 75], the differences observed from 229 ms to 250 ms after IC in this study challenge the notion of a foremost ACL protective strategy.

Second, it is possible that the increased co-activation is the result of individual muscle activation pattern changes to decrease demands on the knee. For example, both the lateral hamstrings and the hip joint contribute more work to SLHD landings in ACL-reconstructed athletes [17]. Movement strategies to reduce knee loads have consistently been reported after ACL-reconstruction [17, 36, 77, 78], and it is possible that increased co-activation is a result of individual muscle actions to direct landing loads away from the knee. Yet, reduced knee flexion moments during the SLHD were reported at peak loading, approximately 100 ms after IC [58]. Consequently, the timing of the observed differences in this study question the relationship between increases in co-activation and reduced knee loading.

Last, another potential reason for increased co-activation might be that it reflects a postural control strategy during the SLHD, which requires maintaining balance after landing [31]. Deficits in postural control have been reported in ACL-reconstructed individuals [79, 80], and postural stability is linked to co-activation through several observations [81, 82]. ACL-injuries can change central nervous system function due to altered proprioceptive input [83–85], leading to higher attentional focus and increased activity in visual-sensory processing networks during motor control and proprioceptive tasks [86–88]. Interestingly, Smeets et al. [31] observed a decrease in thigh muscle co-activation to levels of healthy control under dual-task conditions, where central processing capacity may be limited. Consequently it was hypothesised that central reorganisation, with a consecutive higher attentional and central drive for postural control, is responsible for heightened co-activation [85]. The observed co-activation differences in this study neither directly support nor contradicts this hypothesis. However, considering that postural stabilisation after landing typically extends beyond multiple seconds after IC [89] and that the suprathreshold cluster in our study reached the limit of the analysed time frame, the results suggest that assessment of co-activation during SLHD testing could benefit from longer time-intervals than the 250 ms after IC used in this study.

Regardless of perspective, we would argue that the increased co-activation observed in this study is most likely a compensation for underlying deficits. This could be clinically important. For example, Smeets et al. [31] not only demonstrated that co-activation was reduced under additional cognitive load but also that ACL-reconstructed individuals, in contrast to healthy individuals, did not increase co-activation levels under a perturbation condition. Heightened co-activation during controlled landing could therefore limit the adaptability to more challenging conditions and in this case, should be considered when assessing neuromuscular control. However, this remains highly speculative, and more research is needed to understand the relevance of possible changes in co-activation. Nonetheless, our results add to previous evidence that at one year post-surgery, neuromuscular function does not seem to be restored to normal [46].

### Co-activation between 2D scored landings

This study demonstrates, that a proposed three-point knee flexion scoring system [44] did not differentiate between landings with higher or lower co-activation. Previous research revealed a moderate correlation between thigh muscle co-activity and knee flexion during gait in ACL-reconstructed individuals [30] and single-leg hop landings in healthy people [33]. Other studies have shown that increasing the knee flexion angle during landing decreases co-activation of thigh musculature [35, 90]. The non-significant result in this study is not necessarily contradictory as the categorisation of knee flexion for the scoring system may not have been sensitive enough to reveal existing differences. Further, Podraza and White [35] observed the largest differences between conditions in which knee flexion was restricted to less than or more than 25°, which are uncommon kinematics in the SLHD [17]. In general, the absence of correlation between co-activation and the 2D knee flexion score may signify that knee flexion angle alone is an inadequate representation of the neuromuscular strategies that contribute to landing stability. Co-activation reflects dynamic muscle coordination, whereas peak knee flexion is a single kinematic parameter that may not be sensitive to the temporal complexity of muscle activation patterns.

From a methodological perspective, the 2D knee flexion categorisation may lack the requisite precision to detect subtle neuromuscular differences. The scoring system utilises relatively broad angle intervals (e.g., < 60°, 60–80°, > 80°), which may mask meaningful between-participant variability. Furthermore, the limited number of trials classified within the highest flexion category resulted in diminished statistical power to detect relationships with co-activation. The three-point scoring system was originally tested in a population that included competitive athletes [44]. The population in our study differed and had lower scores on the TAS, which is likely a reason for the lower number of participants reaching knee flexions greater than 80°. Moreover, while AVADS has shown good to excellent agreement with 3D motion capture, it tends to underestimate real sagittal joint angles [42].

Nonetheless, it can be concluded that the three-point, 2D knee flexion scoring system is not suitable for revealing differences in thigh muscle co-activation in a recreational sporting population. The distribution of the scores among legs and groups indicated, however, that the 2D analysis and scoring system could be suitable for detecting differences in landing kinematics after ACL-reconstruction.

### Limitations

Several limitations of this study should be considered when interpreting the results. First, the sample size was determined for a different project that assessed longitudinal changes during rehabilitation. To decrease the probability of false positives, we applied Bonferroni corrections to the alpha levels and chose two-sided tests. However, this study may still lack sufficient power to reliably assess differences one year post reconstruction. Furthermore, several factors contributed to heterogeneity in the study sample. These include the variety of concomitant injuries and diverse levels of sporting activities. Additionally, we did not control the rehabilitation process that the participants underwent. The amount and quality of rehabilitation could affect neuromuscular adaptations and subsequently influence our outcomes. Nonetheless, all ACL-reconstructed participants underwent standard care, reflecting the status one year after ACL rupture and reconstruction in a normal population sample. Moreover, we could not select participants based on a return to sports process. The comparison of TAS scores confirmed that participants on average resumed pre-injury levels of sporting activity, but we did not have information on how participants were cleared to return to sports.

Another limitation is that the submaximal EMG-normalisation procedure does not align with the normalisation to maximal voluntary contraction, as proposed for the CCI by Rudolph et al. [22]. In general, there is no agreement on the optimal normalisation procedure [91]. Nonetheless, EMG signals in the present study were normalised to a low-intensity reference task (walking at 3 km/h), which differs substantially in neuromuscular demands and activation patterns from the high-intensity SLHD task. This discrepancy affects the scaling of muscle activation and, consequently, the estimation of CCI. However, this approach was deliberately chosen to provide a consistent and reproducible reference across participants, as submaximal gait cycles tend to be less susceptible to variability than maximal or highly dynamic tasks. In contrast, normalisation to maximal voluntary contractions or high-intensity tasks may be influenced by performance variability, insufficient familiarisation, or the inability of some participants to produce true maximal efforts [92]. Averaging EMG over multiple gait cycles therefore likely yields a more stable estimate of neuromuscular activity while also encompassing knee flexion angles within a range comparable to those observed during jump landings [16, 93]. Moreover, comparisons of CCIs between groups remain valid and comparison with other studies remain possible, particularly because the denominator corresponded to the same muscle group (quadriceps) as in other commonly used normalisation approaches [40]. Nevertheless, it cannot be excluded that this normalisation strategy underestimates relative activation levels during SLHD, and the findings should therefore be interpreted with consideration of this methodological constraint. 

Last, it is important to note that we assessed muscle co-activation around the knee without considering all muscles relevant to knee stability. For example, the gastrocnemius muscle might play a significant role in knee stability [94, 95]. Moreover, the biarticular rectus femoris muscle can influence both inter-joint coordination and knee joint loading, particularly during eccentric landing phases [69]. Excluding the rectus femoris may therefore have limited the completeness of our assessment of quadriceps activity and could have influenced the calculated co-contraction indices between the anterior and posterior thigh muscles.

## Conclusions

This study revealed that alterations in thigh muscle co-activation during the landing of a single leg hop for distance exist one year after ACL-reconstruction, specifically from 229 ms to 250 ms after IC. This suggests that surgical reconstruction and rehabilitation after ACL-injury do not restore neuromuscular control during landing, and consideration of muscle co-activation could be important during SLHD testing. However, the three-point knee flexion scoring system using 2D movement analysis is not suitable for capturing differences in co-activation. Therefore, other instruments are needed to assess these alterations in neuromuscular control in clinical practice. The large interindividual differences in co-activation introduce a significant degree of uncertainty and further research is crucial to confirm these results, especially the specific time frame in which co-activation alterations are present. Furthermore, the neurophysiological processes that underlie changes in co-activation need to be established to clarify their clinical relevance and improve rehabilitation and assessment of neuromuscular control.

## Supplementary Information

Supplementary Material 1. Additional Files

## Data Availability

The datasets used and analysed in the current study are available from the corresponding author upon reasonable request **.**.

## Abbreviations

ACL: Anterior cruciate ligament
AVADS: Automated Video Analysis of Dynamic Systems
CCI: Co-contraction index
EMG: Electromyography
IC: Initial contact
KOOS: Knee Injuries and Osteoarthritis Outcome Score
RMS: Root mean squares
SLHD: Single leg hop for distance
SPM: Statistical Parameter Mapping
TAS: Tegner Activity Scale
