# Supplementary material for: Thigh muscle co-activation during a single leg hop for distance one year after ACL-reconstruction and its relationship with a 2D knee flexion score: a cross-sectional study
Source: BMC Musculoskelet Disord. 2026 Jul 30;27:940. doi: 10.1186/s12891-026-10236-x (PMC13721325; doi:10.1186/s12891-026-10236-x)
Supplement: Supplementary file 1 [file 12891_2026_10236_MOESM1_ESM.pdf]

Additional Files

1.1. Additional file 1: English translation of the case report form (anthropometric data and sports participation section)

Participant ID<br>Continuous number	☐☐

1 **Inclusion and Exclusion Criteria –** to be completed by the clinical examiner::

 ❑ YES ❑ No<br>❑ YES ❑ No	Inclusion criteria fulfilled:<br>- Age between 18 and 50 years<br>- At least 2 × 45 minutes of physical activity per week
 ❑ YES ❑ NO<br>❑ YES ❑ NO<br>❑ YES ❑ NO<br>❑ YES ❑ NO<br>❑ YES ❑ NO<br>❑ YES ❑ NO	Exclusion criteria (based on medical history) excluded:<br>-Back pain or spinal disorders<br>-Muscular or neurological injuries or disorders<br>-Thrombosis, recent fractures within the last 12 months<br>-Bone tumors, vascular diseases, alcohol dependency, or dementia<br>-Acute infection or cold<br>-Intensive physical exercise on the day before testing

2 **Anthropometric Data**

Sex	❑ male ❑ female
Age (years)	
Body Height	_._ _ m
Body weight	_ _._kg
Leg dominance	❑ left / ❑ right „the leg you would kick a ball with“
Physical activity	❑ yes / ❑ no
If yes, please describe the type and amount of activity per week:	Example: Badminton – 60 minutes<br>1.__________<br>2.__________<br>3.__________<br>4.__________<br>5.__________<br>→ Specify the type of sport or type of physical activity.
Total amount of physical activity per week:	_ _ _ min
Rupture:	Side of rupture:<br>❑ left<br>❑ right

1.2. Additional file 2: Results of individual muscle analysis

Figure 3: Normalised activities of M. Semitendinosus muscle during SLHD landing

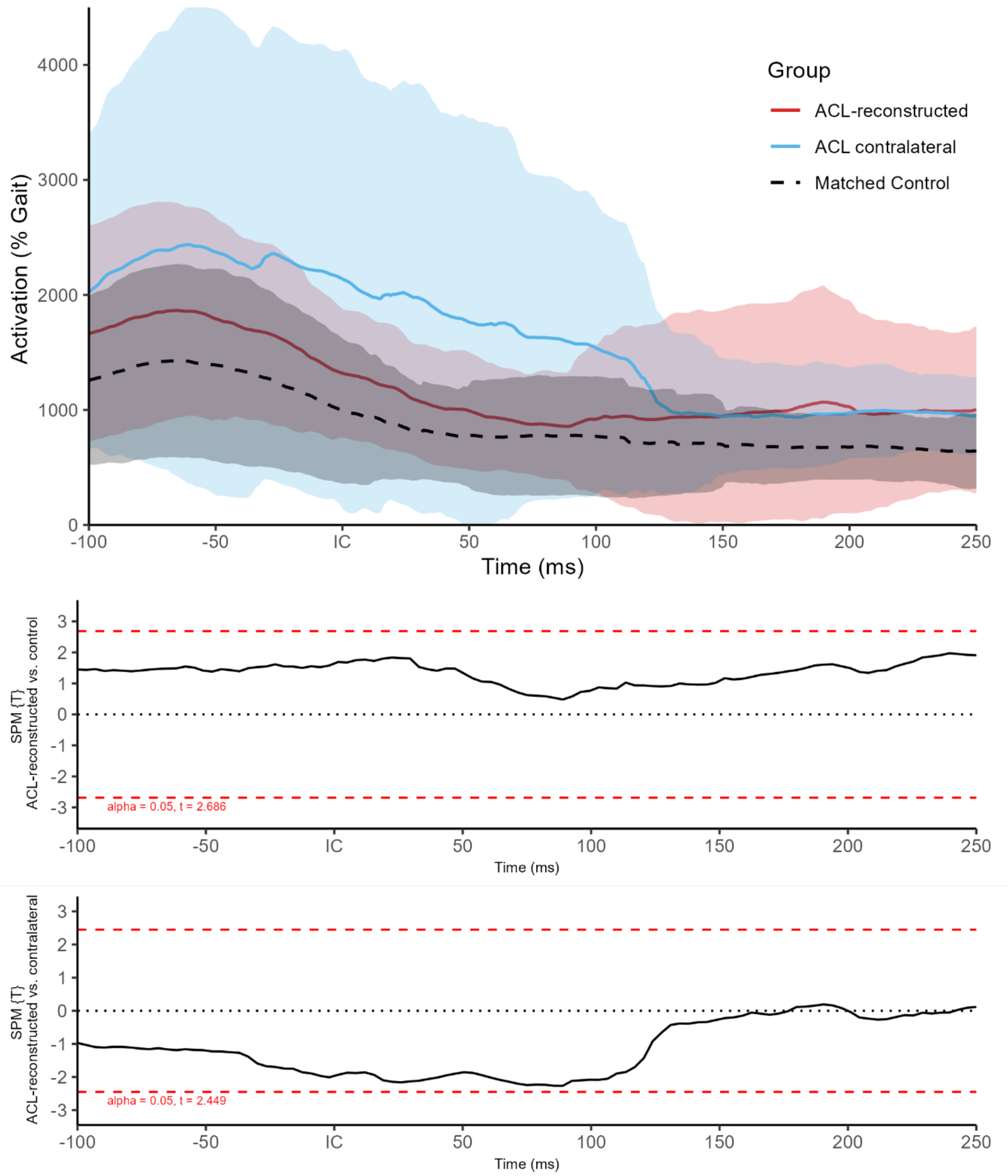

3

Fig. 3. Upper row: average normalised activation of the M. Semitendinosus during landing. Shaded zones represent standard deviations. Middle row: SPM output of nonparametric unpaired t-test comparing ACL-reconstructed legs and matched control legs. Lower row: SPM output of nonparametric paired t-test comparing ACL-reconstructed legs and contralateral legs. If the t-curve (black line) exceeds the critical threshold (red dashed line), the difference is deemed significant.
IC = initial contact; ACL = anterior cruciate ligament

***Figure 4**: Normalised activities of M. Biceps Femoris during SLHD landing*

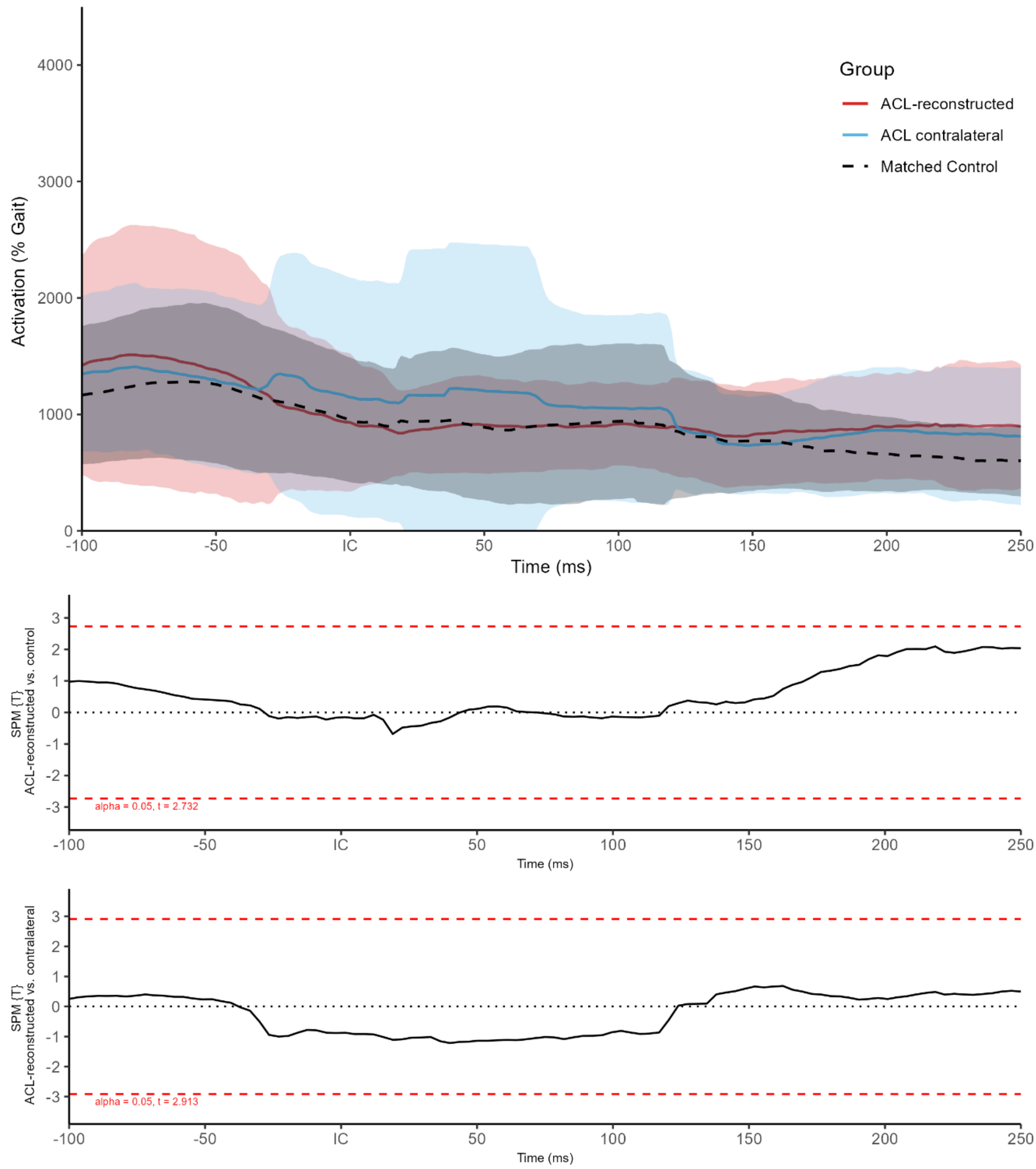

Fig. 4. Upper row: average normalised activation of M. Biceps Femoris during landing. Shaded zones represent standard deviations. Middle row: SPM output of nonparametric unpaired t-test comparing ACL-reconstructed legs and matched control legs. Lower row: SPM output of nonparametric paired t-test comparing ACL-reconstructed legs and contralateral legs. If the t-curve (black line) exceeds the critical threshold (red dashed line), the difference is deemed significant. IC = initial contact, ACL = anterior cruciate ligament

Figure 5*: Normalised activities of M. Vastus Lateralis during SLHD landing*

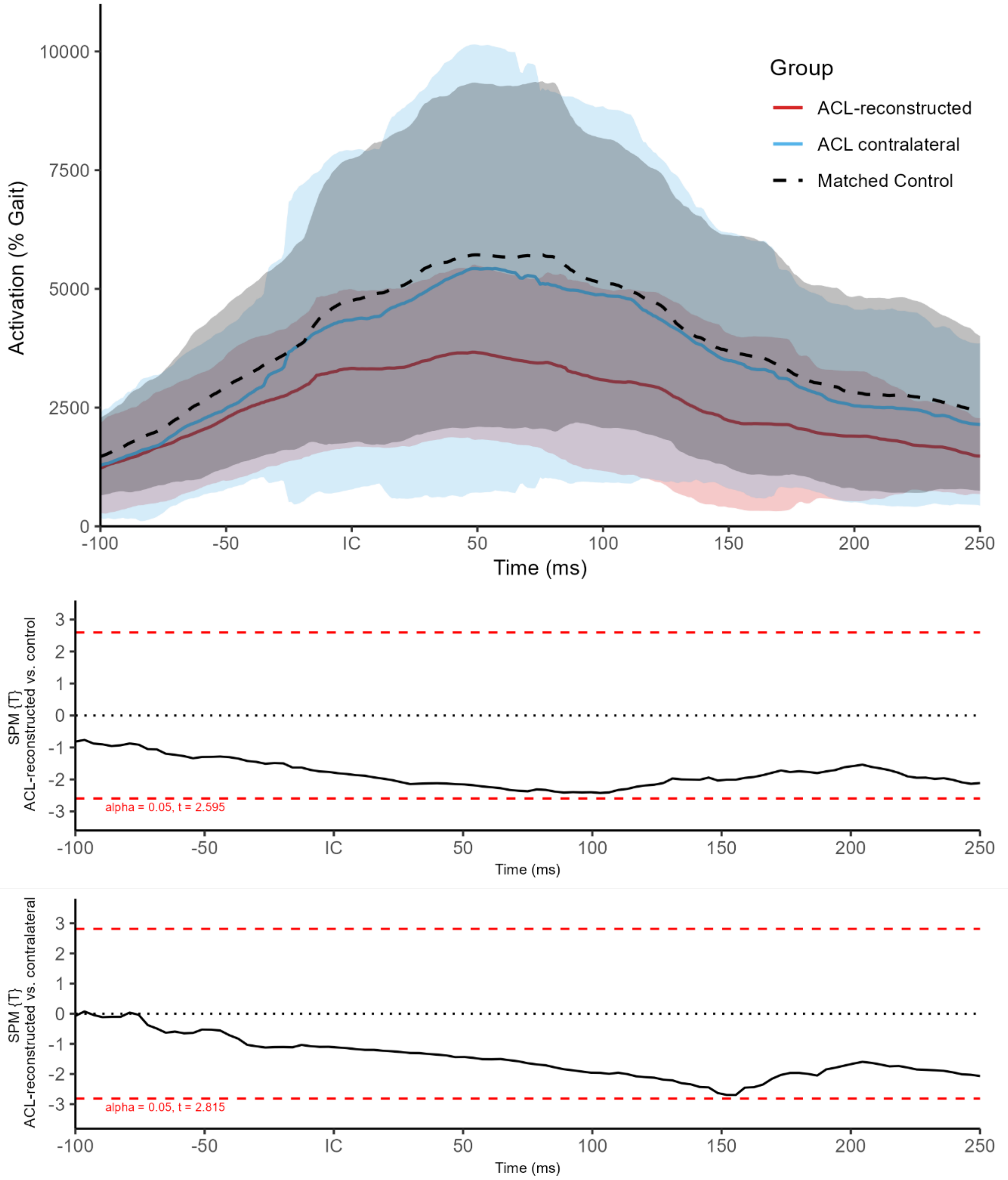

Fig. 4. Upper row: average normalised activation of M. Vastus Lateralis during landing. Shaded zones represent standard deviations. Middle row: SPM output of nonparametric unpaired t-test comparing ACL-reconstructed legs and matched control legs. Lower row: SPM output of nonparametric paired t-test comparing ACL-reconstructed legs and contralateral legs. If the t-curve (black line) exceeds the critical threshold (red dashed line), the difference is deemed significant. IC = initial contact, ACL = anterior cruciate ligament

***Figure 6**: Normalised activities of M. Vastus Medialis during SLHD landing*

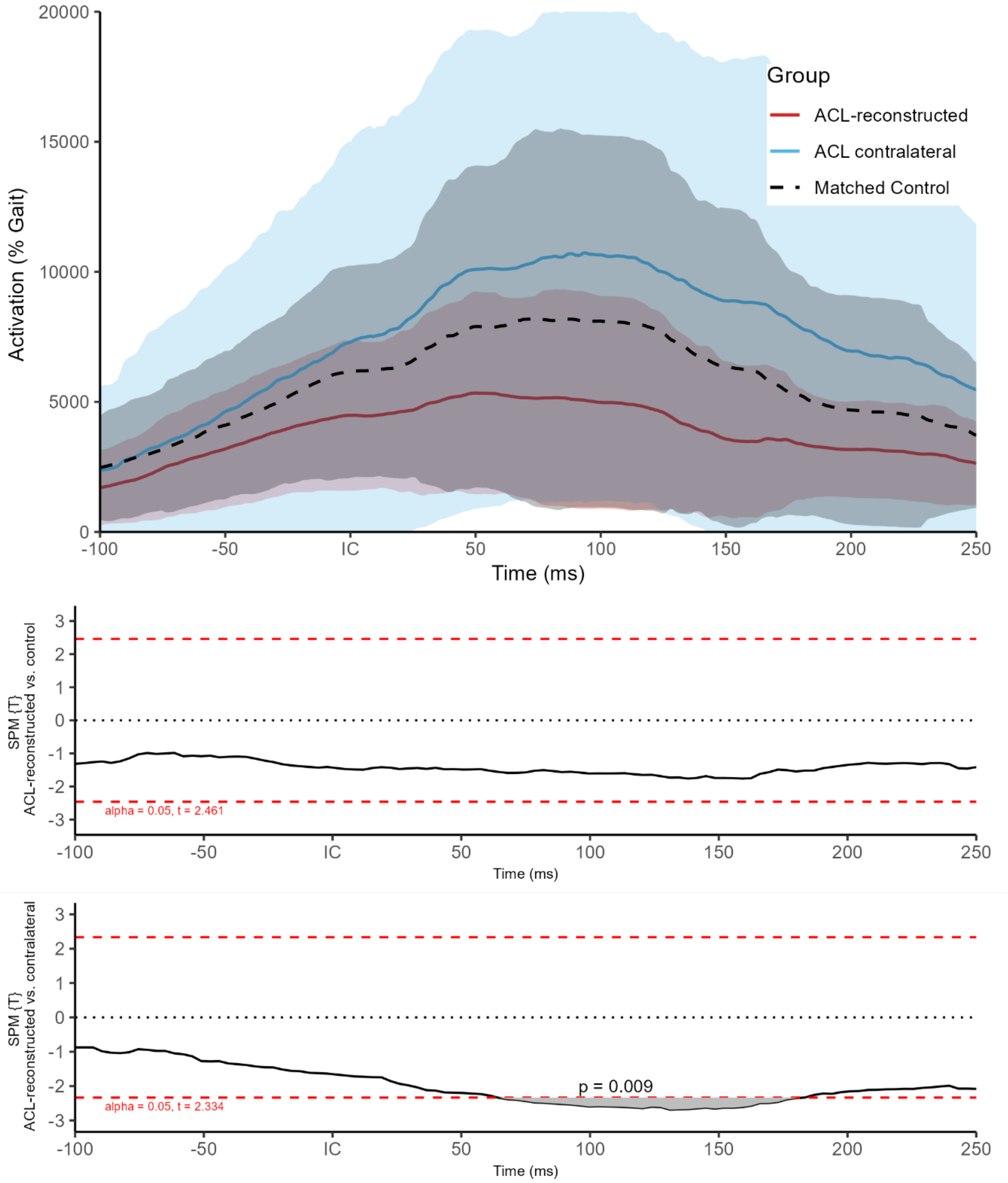

Fig. 4. Upper row: average normalised activation of M. Vastus Medialis during landing. Shaded zones represent standard deviations. Middle row: SPM output of nonparametric unpaired t-test comparing ACL-reconstructed legs and matched control legs. Lower row: SPM output of nonparametric paired t-test comparing ACL-reconstructed legs and contralateral legs. If the t-curve (black line) exceeds the critical threshold (red dashed line), the difference is deemed significant. IC = initial contact, ACL = anterior cruciate ligament

1.3. Additional file 3: Analysis of peak knee flexion between groups and legs

Table 1: Peak Knee Flexion During Landings

Group	Peak knee flexion (mean ± sd)
ACL-reconstructed	47.52°±10.01°
ACL contralateral	56.01°±8.06°
Control matched	59.20°±9.83°
Control contralateral	62.89°±7.72°

Average peak knee flexion for all analysed landings, irrespective of availability of EMG data.
ACL = anterior cruciate ligament, sd = standard deviation

Unpaired Welch's t-test between the ACL-reconstructed leg and matched control leg: p= 0.001; t= -3.43; df = 31.99; CI= (-18.61, -4.74).

Unpaired Welch's t-test between the ACL contralateral leg and control contralateral leg: p= 0.018; t= -2.50; df= 30.32; CI= (-12.51, -1.27).

Paired Welch's t-test between the ACL-reconstructed leg and ACL contralateral legs: p= 0.003; t= -3.37; df= 16.0; CI= (-13.28, -3.01).
